# Correlation of serum CTRP9 and CTRP15 levels with HOMA-IR and HOMA-B in metabolic syndrome patients with and without coronary artery disease

**DOI:** 10.12669/pjms.42.2.12826

**Published:** 2026-02

**Authors:** Fareena Ahmad, Uzma Zafar, Hafiz Abdul Rehman Tariq, Saba Khaliq

**Affiliations:** 1 Dr. Fareena Ahmad, MPhil. Department of Physiology, University of Health Sciences, Lahore, Pakistan; 2 Dr. Uzma Zafar, PhD. Department of Physiology, University of Health Sciences, Lahore, Pakistan; 3 Dr. Hafiz Abdul Rehman Tariq, MPH. Institute of Public Health, Lahore, Pakistan; 4 Dr. Saba Khaliq PhD. Department of Physiology and Institute of Allied Health Sciences, University of Health Sciences, Lahore, Pakistan

**Keywords:** CTRP15, CTRP9, Coronary Artery Disease, Metabolic Syndrome, HOMA-IR, HOMA-B

## Abstract

**Objective:**

This study aimed to evaluate and compare the serum levels of CTRP9, CTRP15, HOMA-IR, and HOMA-B in metabolic syndrome patients, with and without coexisting coronary artery disease.

**Methodology:**

This was a cross-sectional comparative study involving two groups, each with 40 patients. Group-A comprised metabolic syndrome patients with coronary artery disease, whereas Group-B included metabolic syndrome patients without coronary artery disease. The study was carried out from September 20^th^, 2019 to August 31^st^, 2020 at Department of Physiology & Cell Biology, University of Health Sciences, Lahore. After getting written informed consent clinical and biochemical characteristics of the patients were assessed. Data analysis was conducted with IBM SPSS version 26.

**Results:**

The systolic (p=0.012) as well as diastolic (p=0.001) blood pressure and serum low-density lipoprotein cholesterol (p=0.032) was significantly higher in the Group-A when compared with the Group-B. Serum high-density lipoprotein was significantly lower (p = 0.031) in Group-A compared to Group-B. Significantly elevated levels of HOMA-IR (p=0.001), and CTRP15 (p=0.001) were present in Group-A as compared to the Group-B. A statistically significant negative correlation was observed between HOMA-B and CTRP15 serum levels (rho=-0.356, p=0.024) in Group-A.

**Conclusion:**

In this study, decreased insulin secretion was found to correlate with increased CTRP15 levels in patients with metabolic syndrome and coronary artery disease. This finding suggests the potential role of CTRP15 in the pathophysiology of beta cell dysfunction possibly reflecting either a state of CTRP15 resistance or a compensatory response to impaired insulin secretion.

## INTRODUCTION

Metabolic syndrome (METS) is a global health issue defined by a combination of clinical, metabolic, and biochemical disturbances, such as central obesity, elevated blood pressure, insulin resistance, and abnormal lipid profiles. METS and its related components are highly prevalent, with estimated global burden ranging from 12.5% to 31.4%, depending on the criteria used for diagnosis.^1^ Individuals with METS have a threefold higher risk of developing coronary artery disease (CAD) and stroke, and are twice as likely to die from cardiovascular or cerebrovascular conditions compared to those without the syndrome. The pathogenesis of METS includes both genetic and modifiable factors that activates the pro-inflammatory environment leading to CAD and other METS-related adverse events. Timely lifestyle changes and management of risk factors can help to prevent the development of major complications associated with METS, such as Type-II diabetes, CAD, liver disorders, and cognitive decline.^2^

C1q/TNF-related proteins (CTRPs) is a family of proteins, secreted by adipose tissues, heart, skeletal muscles and various organs of the body.^3^ The proper receptors for CTRP function have not been recognized, however, the probable targets are adipose organ, endothelial and hepatic tissue.^4^ CTRP9 and CTRP15 are known as cardiokine. CTRP9 is a multimeric protein, which is mainly expressed in stromal cells and adipose tissue. The modular organization of CTRP9 consists of 4 different domains, and it can be secreted with adiponectin as hetero-oligomers. After binding to AdipoR1 (adiponectin receptor 1) and N-cadherin, CTRP9 activates multiple downstream signaling pathways that contribute to the regulation of glucose and lipid metabolism, cell differentiation and vasodilation. CTRP9 has a protective role against ischemic stroke and myocardial injury following ischemia-reperfusion in an AMPK-dependent manner.^5^,^6^ Although some studies have noted lower CTRP9 levels in people with T2DM, circulating CTRP9 still shows clear positive links with BMI, fasting glucose, insulin, and LDL-C—pointing to its close association with obesity and insulin resistance. Experimental findings further suggest that CTRP9 may help protect against diabetic retinopathy by reducing oxidative stress and apoptosis through the AMPK–Nuclear factor pathway.^7^

CTRP15, also known as myonectin, is a recently identified myokine belonging to the CTRP family. It is primarily secreted by skeletal muscle and plays a key role in regulating systemic lipid metabolism, glucose homeostasis, and energy balance.^8^ Emerging evidence suggests that CTRP15 exerts insulin-sensitizing and anti-inflammatory effects, linking skeletal muscle activity to metabolic health. CTRP15 serves as a key molecular link between skeletal muscle and the liver, contributing to systemic lipid homeostasis.^9^ Studies on animal models depict favorable anti atherosclerotic role of CTRP15.^10^ In humans elevated levels of CTRP15 are reported in coronary artery disease patients. Its secretion rises in hyperglycemia and insulin resistance as a compensatory response to promote fatty acid oxidation and enhance glucose tolerance.^8^ These inconsistent results highlight the need to clarify the role of CTRP15 in different disease conditions like insulin resistance, obesity, T2DM and CAD.^11^ Moreover, research from Pakistan assessing CTRP9 and CTRP15 levels in human population remains limited. Investigating the circulating levels of CTRP9 and CTRP15 may uncover potential biomarkers or therapeutic targets in the management of cardio-metabolic disorders.

The present study aimed to compare serum levels of HOMA-IR, HOMA-B, CTRP9 and CTRP15 in METS patients with and without CAD. It also evaluated the relationship of CTRP9 and CTRP15 with HOMA-IR and HOMA-B in METS patients with and without CAD.

## METHODOLOGY

It was a cross-sectional comparative study carried out at the Department of Physiology & Cell Biology, over a period of one year from September 20^th^, 2019 to August 31^st^, 2020 after the approval of synopsis by the Advanced Studies and Research Board, University of Health Sciences, Lahore.

### Ethical approval:

The study was approved by Ethical review committee of University of Health Sciences, Lahore (UHS/REG-19/ERC/321.9; dated September 27, 2019) Written informed consent was taken from every participant. They were reassured that the records will be kept confidential and anonymous.

The participants were recruited from the Diabetic Center of Sheikh Zaid Hospital, Lahore with convenience sampling technique. The sample size was calculated using World Health Organization sample size calculator keeping the power of study equal to 90% and level of significance 5%. The estimated sample size was a total of 80 patients (40 in each group).^11^ Group-A included cases of METS with CAD and Group-B included cases of METS without CAD.

### Inclusion criteria for Group-A (METS with CAD):

Patients of METS were recruited according to IDF (International Diabetes Federation) guidelines for Asian Indians.^12^ It requires central obesity (waist circumference ≥90 cm in men, ≥80 cm in women) plus any two of the following:

- Elevated triglycerides (≥150 mg/dL),
- Reduced HDL cholesterol (men <40, women <50 mg/dL) or on lipid lowering drugs,
- Raised blood pressure (≥130/85 mmHg) or on antihypertensive,
- Raised fasting glucose (≥100 mg/dL) or on treatment for diabetes mellitus. Patients were included in CAD group, if they had any one or more of the following evidences; a history of ischemic heart disease, angina, or myocardial infarction. ECG abnormalities suggestive of ischemia (such as ST-segment depression, T-wave inversion, Q waves, or arrhythmias), angiographic proof of CAD or abnormal echocardiographic findings (ejection fraction <55% or regional wall motion abnormalities).^13^

### Inclusion criteria for Group-B (METS without CAD):

Age and sex-matched patients were recruited who have METS as per IDF guidelines without CAD evidence.^12^,^13^

### Exclusion criteria for Group-A and B:

All those cases of METS who have evidence of any of the inflammatory and infectious state (affecting the biochemical parameters) like chronic liver disease, end-stage renal disease, neuropathies, chronic infections, malignancies, and autoimmune diseases were excluded.

After getting written informed consent, every individual was assessed by taking history and performing a physical examination. Anthropometric measurements were recorded using standardized procedures. Following an overnight fast of 8-10 hours, five ml of venous blood was collected aseptically. Blood was secured in the vacutainer with clot activator for serum extraction. Blood samples were centrifuged to separate the serum, which was then aliquoted into 1.5 ml Eppendorf tubes and stored at -40°C until analysis.

Blood pressure was measured using a standard mercury sphygmomanometer and stethoscope with the patient in a seated position after at least 5 minutes of rest. Two readings were taken from the right arm at 5-minute intervals, and the average of the two was recorded. Waist circumference was measured over light clothing at the umbilical level, midway between the lower rib margin and iliac crest, using a non-compressive metric tape. Body mass index (BMI) was calculated by measuring the weight and height of each participant. Weight was recorded in kilograms using a calibrated digital scale with the subject wearing light clothing and no shoes. Height was measured using a stadiometer with the participant standing upright against a vertical surface.^14^ BMI was calculated using the formula:^15^

BMI=weight (kg)/height^2^ (m^2^)

Serum insulin concentrations (mU/L) were determined using a commercially available ELISA (Enzyme-Linked Immunosorbent Assay) kit Elabscience. Fasting blood glucose levels (mmol/L) were assessed using a glucometer Accu-Chek, following standardized protocols. Lipid profile components, including serum high-density lipoprotein (HDL) and triglycerides, were analyzed by the colorimetric method with appropriate reagent kits. Insulin resistance was estimated using the HOMA-IR formula, as originally proposed by Matthews and colleagues.^16^ The following formula was used to measure HOMA-IR (Homeostatic Model Assessment for Insulin resistance):

- HOMA-IR=(Fasting plasma glucose (mmol/l)×Fasting plasma insulin (mU/l))/22.5
- Homeostatic Model Assessment of Beta-cell Function (HOMA-B) estimates how effectively the pancreas produces insulin in response to glucose. It was calculated using the following formula.^16^,^17^
- HOMA-β=(20 x Fasting plasma insulin (mU/l))/(Fasting plasma glucose-3.5)
- Serum CTRP9 and CTRP15 levels were measured by Enzyme-Linked Immunosorbent Assay (ELISA) kit manufactured by Elabscience Biotechnology Inc, respectively. The coefficient of variation was less than 10%.

### Statistical Analysis:

The data were analyzed using SPSS version 26. Normality was assessed with the Shapiro-Wilk test, which showed that only age was normally distributed (p > 0.05). Age was presented as mean ± Standard deviation and compared using the independent-samples t-test. All other variables, being non-normally distributed, were expressed as median with interquartile range (IQR) and analyzed using the Wilcoxon Rank Sum test. Spearman correlation was used to assess the association of HOMA-IR and HOMA-B with CTRP9 and CTRP15.

## RESULTS

The study included a total of 80 patients; 40 diagnosed with METS and CAD (Group-A), and 40 with METS without CAD (Group-B). In Group-A, 29 (72.5%) males and 11 (27.5%) females were part of the study. In Group-B, 34 (85%) were males, and the rest 6 (15%) were females. The systolic and diastolic blood pressure was significantly higher in the Group-A when compared with Group-B (P=0.006). (Table-I). Comparison of biochemical parameters is given in Table-II.

**Table-I:** Comparison of anthropometric variables between metabolic syndrome patients with and without coronary artery disease.

Study Variables	Metabolic Syndrome with CAD [n=40]	Metabolic Syndrome without CAD [n=40]	P-value
Age (in years)	45.70 ± 1.32	46.60 ± 1.45	0.648
Systolic Blood Pressure (mm Hg)	130.0 (120.0-140.0)	120.0 (110.0-130.0)	0.012*
Diastolic Blood Pressure (mm Hg)	90.0 (80.0-90.0)	70.0 (70.0-90.0)	0.001*
Body Mass Index (kg/m^2^)	29.88 ± 0.82	28.60 ± 0.61	0.340
Waist Circumference (cm)	100.60 ± 1.66	101.18 ± 1.43	0.944

**Table-II:** Comparison of biochemical parameters between metabolic syndrome patients with and without coronary artery disease.

Biochemical parameters	Metabolic Syndrome with CAD [n=40]	Metabolic Syndrome without CAD [n=40]	P-value
Fasting serum glucose (mmol/l)	8.5 (7.2-11.8)	6.8 (6.0-8.2)	0.007*
Fasting insulin (µIU/ml)	30.2 (20.9-48.5)	19.0 (11.2-35.4)	0.021*
HOMA-IR	13.7 (7.8-19.2)	6.6 (3.3-15.0)	0.001*
HOMA-B	4.9 (3.3-12.1)	5.8 (3.3-8.7)	0.493
Serum HDL (mg/dl)	36.78 ± 1.34	40.63 ± 1.13	0.031*
Serum triglycerides (mg/dl)	200.5 (148.0-248.5)	165.0 (133.0-240.8)	0.601
Serum LDL-C (mg/dl)	114.5 (90.0-130.0)	87.0 (56.0-122.3)	0.032*
Serum Cholesterol (mg/dl)	192.5 (166.0-223.0)	213.0 (180.0-242.0)	0.291
CTRP9 (ng/ml)	0.2 (0.1-0.3)	0.2 (0.1-0.3)	0.273
CTRP15 (ng/ml)	43.0 (40.7-46.9)	40.4 (38.1-43.3)	0.001*

A Spearman correlation was applied to determine the relationship between HOMA-IR, HOMA-B, CTRP9, and CTRP15. There was a strong, negative correlation between HOMA-B and CTRP15 level, which was statistically significant (rho= -.356, p= 0.024). However, this trend was only observed in patients of the METS with CAD. No other significant correlation was observed in any of the other indicators in all of the patients with or without CAD (Table-III).

**Table-III:** Correlation of HOMA-IR and HOMA-B with CTRP9 and CTRP15.

All Patients (N=80)	*Variables*	*HOMA-IR*	*HOMA-B*
		*Rho value*	*Rho value*
		*P value*	*P value*
	CTRP9	0.059	-0.105
		0.601	0.355
	CTRP15	0.023	-0.016
		0.837	0.355
Metabolic syndrome with CAD (n=40)	CTRP9	0.013	-0.178
		0.936	0.271
	CTRP15	-0.098	-0.356
		0.0548	0.024*
Metabolic syndrome without CAD (n=40)	CTRP9	0.065	-0.049
		0.692	0.762
	CTRP15	-0.059	0.236
		0.717	0.142

An independent sample t-test was used to evaluate the p-value of age. Wilcoxon rank sum-test was employed to calculate the rest of the p-values. *A p-value of less than 0.05 was considered to be statistically significant. Data are represented as mean ± standard deviation in case of normal distribution. Median (IQR) is represented where data distribution is not normal. “n” represents the number of cases in each group. CAD (coronary artery disease).

Wilcoxon rank sum-test was employed to calculate the p-values. *A p-value of less than 0.05 was considered to be statistically significant. Median (IQR) is given as data distribution is not normal. “n” represents number of cases in each group. CAD (coronary artery disease).

Spearman correlation was applied to evaluate the association of HOMA-IR and HOMA-B with CTRP9 and CTRP15 among (1) all the patients; (2) Metabolic syndrome with CAD patients; (3) Metabolic syndrome without CAD patients. *A p-value of less than 0.05 is considered statistically significant. CAD (coronary artery disease).

## DISCUSSION

The objectives of this study were to compare and correlate serum levels of CTRP9, CTRP15, HOMA-IR and HOMA-B in METS patients with and without CAD. In the present work CTRP15, levels and HOMA-IR were considerably higher in individuals with METS and CAD compared to those with METS but without CAD. This result is in concordance with the previous study conducted upon angiographically confirmed CAD patients which reported significantly higher CTRP15 levels in CAD patients as compared to the healthy ones.^18^ However, another study revealed significantly low levels of this protein in CAD patients and CTRP15 levels show a decreasing trend from triple to single vessel disease.^19^ Low CTRP15 level was taken as an independent risk factor for CAD in this study. Various other studies conducted on type2 diabetics have revealed significantly higher CTRP15 levels as compared to the non- diabetics.^18^,^20^ Due to diversity in the results on literature survey, this cardiokine is an emerging field of research with variable roles in metabolic and vascular health. CTRP15 a newly identified adipokine is a paralogue of adiponectin and has been shown to exert beneficial effects on glucose and lipid metabolism. CTRP15 influences metabolic pathways by interacting with adipose organs and skeletal muscles.^21^ Elevated CTRP15 levels in patients with both METS and CAD in our study may reflect a compensatory or protective response to increased metabolic and cardiovascular stress.^18^

In chronic inflammatory conditions such as METS and CAD, CTRP15 may be upregulated to counteract vascular inflammation, insulin resistance and endothelial dysfunction. The role of CTRP15 in diabetes and METS remains unclear, with studies showing both elevated levels linked to insulin resistance and reduced levels associated with diabetes, and polycystic ovary syndrome.^22^ HOMA-IR is a validated surrogate marker of insulin resistance. Its elevation in METS patients with CAD indicates that insulin resistance is more pronounced in those who have developed cardiovascular complications. This supports the established role of insulin resistance as a central pathogenic mechanism linking METS to atherosclerosis and cardiovascular disease.^23^ HOMA-IR and METS have been identified as independent predictors of CAD risk in another study.^24^

In our study, blood pressure, insulin resistance (HOMA-IR), and LDL levels were significantly higher in METS patients with CAD compared to those without CAD, while HDL levels were significantly lower in the CAD group. This pattern is consistent with the pathophysiological progression of METS to overt cardiovascular disease. Elevated blood pressure increases shear stress on vascular walls, promoting endothelial dysfunction and atherosclerosis. Higher insulin resistance reflects greater metabolic dysfunction, which not only impairs glucose uptake but also promotes inflammation and dyslipidemia. Elevated LDL contributes directly to atheroma formation, a key factor in CAD development. Conversely, reduced HDL impairs reverse cholesterol transport, decreasing the body’s ability to clear excess cholesterol from peripheral tissues and arterial walls.^25^ These derangements in metabolic pathways likely exacerbate vascular injury, plaque formation, and chronic inflammation, thereby increasing the risk and severity of CAD in METS patients.^26^

The second main objective of the present study was to evaluate the correlation of HOMA-IR and HOMA-B with CTRP9 and CTRP15. Our results exhibited an inverse correlation of CTRP15 with HOMA-IR and HOMA-B. The correlation of CTRP15 with HOMA-IR was non-significant. However, there was a significant negative correlation between CTRP15 and HOMA-B values in the METS-CAD group. HOMA-B estimates pancreatic beta cell activity based on fasting glucose and insulin levels. A lower HOMA-B indicates impaired insulin secretion, often due to beta-cell dysfunction-a key contributor to the progression from insulin resistance to Type-II diabetes.^2^ This inverse relationship highlights that in patients with advanced metabolic disease (METS-CAD), beta-cell failure is a key pathological event, and CTRP15 may serve as a biomarker reflecting this progression.^27^ In metabolic diseases, resistance to regulatory hormones (e.g., insulin, leptin, adiponectin) is a hallmark. CTRP15, though less studied, is known to have insulin-sensitizing, anti-inflammatory, and metabolic regulatory roles in experimental models.

In this study, CTRP9 levels did not differ significantly between METS patients with and without CAD. Similarly, we were unable to find any significant correlation of HOMA-IR with CTRP9 and CTRP15. It is important to consider here, that some patients in this study were receiving treatment for diabetes, hypertension, and dyslipidemia with medications such as metformin, ACE inhibitors, and statins, which are known to influence cytokine release, insulin resistance, and overall metabolic control.^28^ These confounding factors may have masked or attenuated potential associations between HOMA-IR and CTRP9/CTRP15, highlighting the need for further studies in more subjects with minimal therapeutic interference.

Measuring circulating CTRP9 and CTRP15 levels could have important clinical and translational implications. Since these adipokines are closely linked to insulin resistance, glucose regulation, and beta-cell function, tracking their levels may help identify individuals at risk for metabolic disorders. Additionally, they could provide valuable insights into disease progression and responses to therapy, supporting more personalized approaches to improving metabolic health.

### Limitations and future recommendations:

One of the limitations of the study was potential confounding effects of concurrent medications, which may have influenced the findings. Secondly, the study design was cross-sectional, which limits the ability to establish causal relationships between CTRP15 levels and beta-cell function. Longitudinal studies should be conducted to assess whether changes in CTRP15 levels predict the progression of beta-cell dysfunction, or development of cardiovascular complications at the molecular level. If CTRP15 plays a causal role, targeting its pathway could offer novel therapeutic strategies to preserve β-cell function and prevent progression of vascular inflammation in metabolic syndrome.

## CONCLUSION

CTRP15 levels were significantly higher in patients of METS with CAD as compared to those with METS and without CAD. There was significant negative correlation of CTRP15 with HOMA-B in METS with CAD. The rise in CTRP15 levels alongside declining beta-cell function suggests that CTRP15 may act as a biomarker indicating disease progression or reflect a paradoxical response-where elevated CTRP15 fails to preserve beta-cell function, possibly due to target tissue resistance. The role of CTRP15 in diabetes and METS remains unclear, with studies reporting both elevated and reduced levels in various metabolic conditions. Further research is needed to clarify its function.
